# Interface Analysis of the Complex between ERK2 and PTP-SL

**DOI:** 10.1371/journal.pone.0005432

**Published:** 2009-05-08

**Authors:** Mihaela C. Balasu, Laurentiu N. Spiridon, Simona Miron, Constantin T. Craescu, Axel J. Scheidig, Andrei-Jose Petrescu, Stefan E. Szedlacsek

**Affiliations:** 1 Department of Enzymology, Institute of Biochemistry, Bucharest, Romania; 2 Department of Organic Chemistry , University POLITEHNICA, Bucharest, Romania; 3 Department of Bioinformatics and Structural Biochemistry, Institute of Biochemistry, Bucharest, Romania; 4 Institut Curie Centre de Recherche, Orsay, France; 5 INSERM U759, Orsay, France; 6 Zoologisches Institut, Strukturbiologie/ZBM, Christian-Albrechts-Universität Kiel, Kiel, Germany; Institut de Pharmacologie et de Biologie Structurale CNRS 205, France

## Abstract

The activity of ERK2, an essential component of MAP-kinase pathway, is under the strict control of various effector proteins. Despite numerous efforts, no crystal structure of ERK2 complexed with such partners has been obtained so far. PTP-SL is a major regulator of ERK2 activity. To investigate the ERK2–PTP-SL complex we used a combined method based on cross-linking, MALDI-TOF analysis, isothermal titration calorimetry, molecular modeling and docking. Hence, new insights into the stoichiometry, thermodynamics and interacting regions of the complex are obtained and a structural model of ERK2-PTP-SL complex in a state consistent with PTP-SL phosphatase activity is developed incorporating all the experimental constraints available at hand to date. According to this model, part of the N-terminal region of PTP-SL has propensity for intrinsic disorder and becomes structured within the complex with ERK2. The proposed model accounts for the structural basis of several experimental findings such as the complex-dissociating effect of ATP, or PTP-SL blocking effect on the ERK2 export to the nucleus. A general observation emerging from this model is that regions involved in substrate binding in PTP-SL and ERK2, respectively are interacting within the interface of the complex.

## Introduction

The MAP Kinase pathway represents one of the most important signaling systems in eukaryotes. It controls a large variety of fundamental cellular processes as proliferation, differentiation, cell survival and apoptosis. Various extracellular stimuli, as growth factors, cytokines, mitogens, environmental stress factors can activate this pathway. Following MAP kinase pathway activation, different cytosolic, membrane-bound or nuclear proteins are expressed, which in turn regulate the transcription of specific genes [1]. The best-studied representatives of the MAP kinase family are ERK 1 and 2. Their activation involves phosphorylation of Thr183 and Tyr185 on the activation loop, which promotes reconstitution of the kinase active site [2]. Upon activation, approximately half of ERK2 is translocated to the nucleus [3], where it phosphorylates specific nuclear targets.

PTP-SL is a major regulator of ERK 1/2 MAP kinase activity. There is an unusual reciprocal interaction within the complex formed between these two proteins: ERK 1/2 phosphorylates Thr 253 of PTP-SL and PTP-SL dephosphorylates the regulatory phosphotyrosine (pTyr 185) on the activation loop of ERK 1/2. Dephosphorylation of pTyr 185 causes inactivation of ERK1/2 and its retention in the cytoplasm [4], [5]. Also, association with ERK1/2, phosphorylation of Thr 253 on PTP-SL, as well as dephosphorylation of pTyr 185 on ERK1/2, are strictly dependent on a novel, 16 amino acids long, Kinase Interaction Motif (KIM). This motif is situated between residues 224–239 of PTP-SL and is highly conserved among all members of the PTP-SL sub-family. Within the KIM sequence, there is a PKA consensus phosphorylation motif, Ser 231, which may be phosphorylated and, thus, binding of ERK2 to PTP-SL is impaired [6]. Consequently, the sub-family of PTP-SL seems to control the activity of MAP kinases and mediates the crosstalk between the MAP kinase cascade and the cAMP-dependent kinases. The highly conserved KIM sequence helps binding of all members of the KIM-containing PTPs (PTP-SL, PTPBR7, STEP and HePTP). The sequence C-terminally adjacent to KIM, termed kinase-specificity sequences (KISs), provides binding specificity to ERK1/2 and p38. Thus, PTP-SL preferentially binds ERK1/2 whereas STEP and HePTP selectively bind p38α [7].

Crystal structures of inactive and active forms of ERK2 have been reported [8], [2]. Further crystallographic analysis of inactive form of ERK2 in complex with a 16-mer KIM peptide derived from hematopoietic PTP (HePTP) has shown that peptide binding induces local and long-range conformational changes. The overall aspect of peptide-bound ERK2 is rather similar to the phosphorylated (active) form of ERK2 [9]. Interestingly, crystal structures of other two MAPKs, p38 and JNK1, in complex with docking motif (or D motif – a generical term used for KIM) peptides also revealed peptide-induced conformational changes but which are unique to each enzyme [10], [11]. Similarly, docking specificities were demonstrated for the complexes of Fus3 – a yeast MAPK – with specific peptides as well as for the complex of ERK2 with a KIM peptide from MAP kinase phosphatase 3 [12], [13].

Despite the relatively numerous reports concerning the docking interaction between MAPKs and specific peptides, there are no structural analyses extended to the interactions of sequences proximal to the KIM region as well as to those involving the kinase active sites. On the other hand it is obvious that the interaction between a MAPK and a protein interactor is not limited to the docking interaction. Specifically, no crystal structure of ERK2 in complex with a substrate or other effector proteins, has been so far reported. Consequently, any finding in this respect would be particularly important in understanding the highly specific nature of ERK2 interactions and the rigorous regulation of its activity. Regarding the ERK2 - PTP-SL complex we previously provided structural support for that the kinase action of ERK2 and the phosphatase activity of PTP-SL involve two distinct conformational arrangements of the interacting partners [14]. Details of these conformations and of how the transition between them takes place are yet unknown. The overall structure of the N-terminal region (aa 147–253) of cytosolic PTP-SL domain remains also elusive as the largest PTP-SL catalytic region crystallized so far was 254–549 [14] and the propensity for intrinsic disorder of the 147–253 region is very high. Given that the interacting KIM motif is located here it is reasonable to presume that this region might become structured only in the presence of ERK2.

In this report we investigate the ERK2−PTP-SL complex by isothermal titration calorimetry and chemical cross-linking combined with MALDI-TOF. The resulting experimental data, along with all the rest of structural data existing at hand in the literature are introduced as imposed constraints to build a 3D-model of the ERK2−PTP-SL complex. The model is built starting from the crystal structures of ERK2 and PTP-SL [12], [14] by docking combined with homology modeling and *ab-initio* modeling in regions lacking template, followed by simulated annealing optimization. This provides the contact interface in a conformation consistent with PTP-SL phosphatase activity. The final refined version of the model accounts for the structural basis of several reported observations, and also pinpoints novel characteristics of the interaction interface for this physiologically important complex.

## Results

The overall strategy of the experiments was the following: initially, optimal cross-linking reaction conditions for the complex of ERK2 with PTP-SL were established, then the major cross-linked product was isolated, digested with trypsin and the mixture of peptides was analyzed by MALDI-TOF. In parallel, a control experiment with the non-covalent complex (not treated with cross-linker) was subjected to a similar treatment. Identification of mass spectrometric signals corresponding to cross-linking of a peptide from ERK2 with another one from PTP-SL grounded the empirical constraints imposed in the ERK2−PTP-SL model building. A similar strategy was successfully applied for determination of structural models of other protein complexes and multidomain proteins [15], [16]

### Cross-linking of ERK2 to PTP-SL

**Figure 1:**
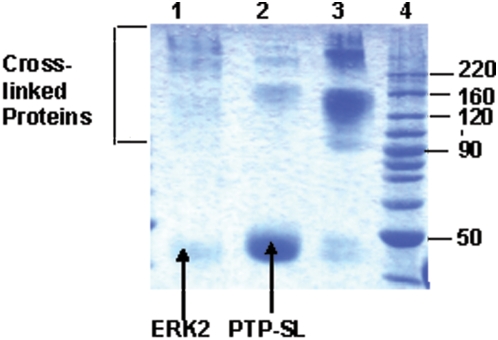
BS^3^ cross-linking reaction specificity. To compare reaction specificity the cross-linking reaction with BS^3^ was performed in parallel with equimolar quantities of ERK2 (lane 1), PTP-SL (lane 2) and ERK2−PTP-SL complex (lane 3). Reaction mixtures were analysed by SDS-PAGE (12,5%).

Two common cross-linking agents were tested: bis(sulfosuccinimidyl) suberate (BS^3^) - a homobifunctional agent reacting with amino groups and providing a linker of 12 carbon atoms - and a heterobifunctional agent producing zero length linker - 1-ethyl-3-(3-dimethylaminopropyl) carbodiimide (EDC) (in presence of N-hydroxysuccinimide (NHS)). All cross-linking experiments were performed in absence of ATP in order to favor the conformations of the complex which enable phosphatase activity of PTP-SL on ERK2 and not the kinase activity of ERK2. Various conditions of the cross-linking reaction were tested for both agents, by modifying concentrations of cross-linker, complex concentration, incubation time and temperature. EDC did not yield significant amount of cross-linked product under any tested reaction conditions (data not shown). Instead, BS^3^ was found to be a relatively efficient cross-linking agent of the ERK2–PTP-SL complex. BS^3^ concentration and incubation period were the most important parameters for the cross-linking yield and composition of the cross-linked reaction products (data not shown). In all situations, a minor band was observed, corresponding to a cross-linked product of approximately 90 kDa as well as another electrophoretic band, corresponding to the major product, of about 120–160 kDa. To analyze whether the cross-linked products thus obtained are homo- or heteromers, cross-linking reactions were performed in parallel, using either individual ERK2 and PTP-SL proteins or the complex of these two proteins. The complex produced a much higher amount of cross-linked product than the individual proteins (FIG. 1). However, homomers of PTP-SL (around 120 kDa) and of ERK2 (mainly over 200 kDa), are also observed (theoretical MWs of PTP-SL and ERK2 are 45623.15 and 42272.61, respectively). Altogether, these results demonstrate that the ratio of homomers in the cross-linked mixture is relatively low as compared to the heteromers, thus proving that cross-linking is strongly favored by the ERK2–PTP-SL complex formation. It should be underlined that, for technical reasons, the inactive form of ERK2 was used in cross-linking experiments, based on the finding that both, active and inactive ERK2 form tight complexes with PTP-SL [4]. One of the difficulties encountered in our attempts to use the active (dephosphorylated) form of ERK2 in cross-linking experiments was that it could not be completely separated from monophosphorylated forms of ERK2; thus, the heterogeneity of the ERK2 preparation would involve additional complications in processing the cross-linking data.

### Purification of the major cross-linked product

**Figure 2:**
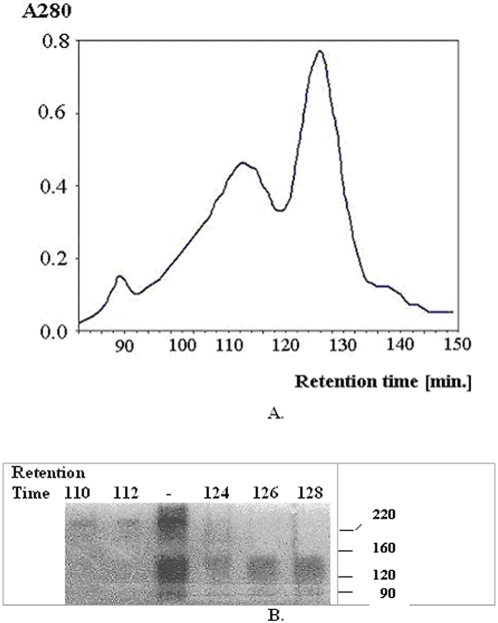
Purification of major cross-linking product of the ERK2−PTP-SL complex. (A) Separation of the reaction products formed by BS^3^ cross-linking of the ERK2−PTP-SL complex was performed by size exclusion cromatography. (B) Fractions obtained through the chromatographic separation of the cross-linking reaction mixture (lanes 1–2, 4–6) were analysed by SDS-PAGE (12,5%). The non-fractionated reaction mixture of BS^3^ cross-linked ERK2−PTP-SL complex was applied on lane 3. Staining was performed with Zn-imidazole reagent.

This step aims at isolating the main cross-linked product and, also to eliminate the non-cross-linked products, the complex molecules linked by disulfide bridges and the monomers. The cross-linking reaction mixture obtained under optimal conditions was applied and run through a Superdex 200 HR gel chromatography column equilibrated with a buffer containing both SDS and DTT. As shown in FIG. 2 three important peaks were obtained. The evaluation of the MWs in selected fractions was performed using SDS-PAGE combined with Zn-imidazole staining, known to confer a more sensitive detection. The resulting electrophoretic pattern shows that the first, minor peak, corresponds to cross-linked products of MWs over 220 kDa, while the second peak corresponds to the major cross-linked product at a MW of 120–160 kDa, in agreement with estimations obtained from SDS-PAGE shown in FIG. 2.

### MALDI-TOF analysis of the cross-linked product

**Figure 3:**
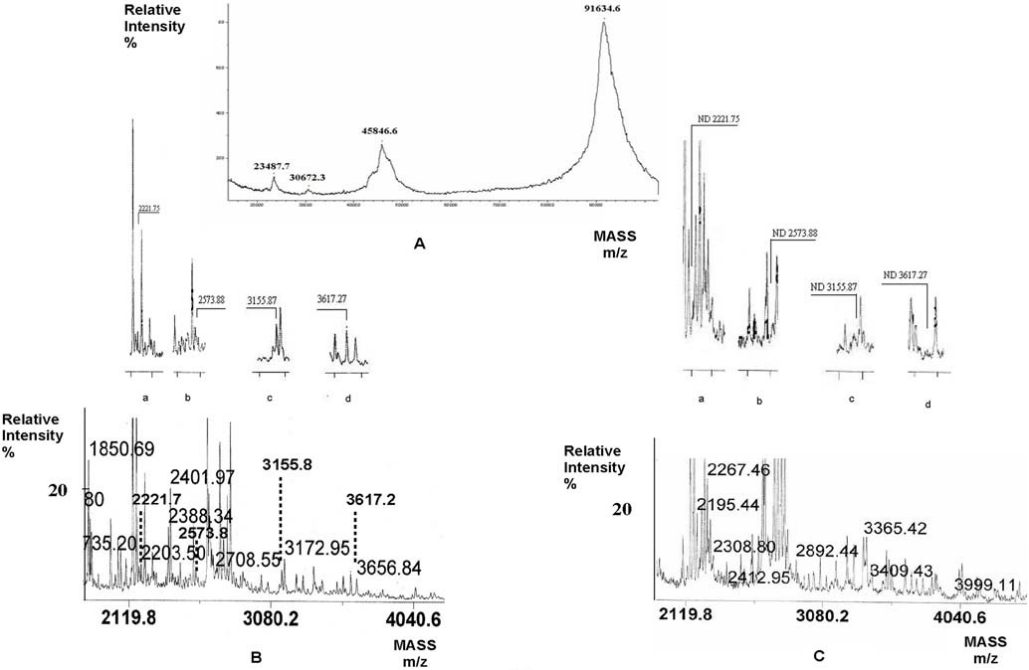
MALDI-TOF spectrum of non-digested and trypsin digested, cross-linked ERK2−PTP-SL complex. (A)The BS^3^ cross-linked complex was purified by size exclusion chromatography, concentrated, SDS removed and finally analysed in presence of sinapinic acid matrix. Representative data for the digested cross-linked (B) and digested non-crosslinked (C) complex are shown. The magnified images a, b, c, d show the four identified tryptic fragments which contain Lys-Lys cross-linked peptides.

**Figure 4:**
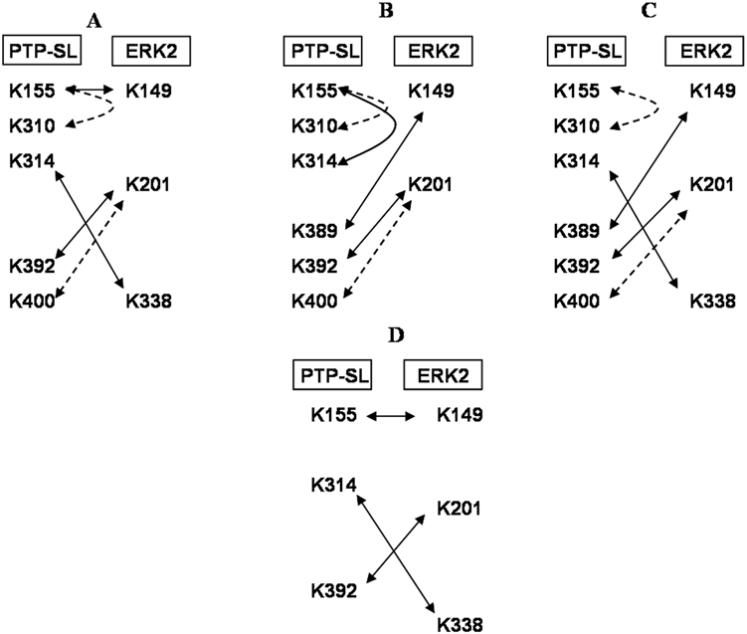
Potential networks of cross-linked lysine residues within the complex between ERK2 and PTP-SL. Processing the MALDI-TOF data resulted from trypsinolysis of the cross-linked complex, three possible networks are revealed (denoted A,B and C). Arrows connect cross-linked lysine residues from PTP-SL and ERK2. Dashed line arrow stands for: (i) an alternative involvement of K400 instead of K392 from PTP-SL in cross-linking K201 from ERK2 and (ii) for a potential cross-link between K155 to K310 of PTP-SL, supporting the signal at *m/z* value of 2221.75. Most probable network (see the text) of cross-links is illustrated in D.

Direct analysis of the cross-linked product by MALDI-TOF mass-spectrometry provided *m/z* values corresponding to PTP-SL monomer and a charged ion peak at 91634.6 which may correspond to a PTP-SL homodimer (FIG. 3A). Lack of signal for the major cross-linked product at 120–160 kDa is not surprising since high MW species sometimes are not detectable by MALDI-TOF [17]. To obtain more information concerning the amino acids at the interface, the purified complex corresponding to the fractions with retention times 126–128 (FIG. 2) was subjected to trypsinolysis and the resulting peptides were analyzed by MALDI-TOF. In parallel, similar analyses were performed, using the non-cross-linked complex. To identify those peptides which were present in the cross-linked product but not in the non-covalent complex, further comparative evaluation of the mass-spectra thus obtained was performed. Initial processing of the spectral data was performed using the Search-X-Link server [18], [19] leading to a list of potential attributions for the signals detected in MALDI-TOF spectra for the cross-linked product. Then, signals having similar *m/z* values in spectra corresponding to non-cross-linked complex, were eliminated. Other signals, corresponding to peptides not involved either in inter- or intramolecular cross-linking, as well as peptides formed by trypsin autolysis were also eliminated. Many of the peptides which should theoretically result from ERK2 and PTP-SL hydrolysis were identified in the mass-spectra. Analysis of mass-spectra obtained on the basis of several independent experiments, evidenced four molecular ions, having the *m/z* values 2221.75; 2573.88; 3155.87 and 3617.27, which are expected to be involved in the cross-linking process (FIG. 3B). Figure 3C shows that these four signals are missing in mass-spectra of the non-cross-linked complex, Table 1 summarizes the theoretical and experimental masses of peptides involved in intra- and intermolecular cross-linking of different amino acidic residues within the complex, and their potential assignments. Two main types of possible cross-linking interactions may be observed: those involving only peptides from PTP-SL and interactions between one peptide from PTP-SL and one from ERK2. It should be noticed that lysine K310 of PTP-SL is involved in three potential sequence assignments, all originating from PTP-SL. Yet, the structure of PTP-SL (PDB code 1JLK) shows that this residue is deeply buried in the core of the molecule being rather inaccessible to the solvent (less than 10% accessibility as measured with NACCESS). Thus, it is quite improbable that the cross-linking agent BS^3^ could have accessed this residue; therefore, assignments involving K^310^ of PTP-SL were eliminated. In this way, the experimental mass of 3155.87 will be assigned to a unique pair of cross-linked peptides. Specifically, this signal corresponds to the cross-linking of the 390–401 fragment of PTP-SL with the 193–205 fragment of ERK2. The first peptide belongs to the surface exposed loop, linking the secondary elements of PTP-SL, ß4 and ß7 [14]. As it contains two lysine residues (K392 and K400) it is not possible to establish which of these residues is directly involved in the cross-linking reaction through BS^3^. The second peptide involved in cross-linking (fragment 193–205) is part of the linker L12 connecting subdomains VIII and IX and is immediately following the P+1 binding area of ERK2 [8], [2]. For the mass spectrometric signals at *m/z* of 2221.75, 2573.88 and 3617.27, there are two alternative assignments and, apparently, this fact generates a large number of possible networks of interacting lysine residues. However, taking into account that only six lysine residues of PTP-SL and three of ERK2 are involved in cross-linking and that a given lysine residue can only be involved in a single cross-linking interaction, it can be inferred that only three alternatives are possible as concerning the networks of the cross-linked lysine residues (FIG. 4). In order to decide the most probable of cross-linking network alternatives mentioned in FIG. 4 we used the unrefined docking model obtained using crystal structures of PTP-SL and ERK2, by positioning the phosphotyrosine of ERK2 lip into the active site of PTP-SL (see also [14]). In this docking model we measured the distance between the C_β_ atoms of K389^PTP-SL^ and its potential cross-linked partner K149^ERK2^ involved both in alternative B and alternative C. That distance was found to be 24 Å, significantly higher than the 21.3 Å limit corresponding to the maximally allowed distance between C_β_ atoms of two lysines cross-linked with BS^3^ cross-linker. Therefore, this cross-linking interaction is highly improbable and consequently, alternatives B and C can be eliminated. The other two distances between lysine residues involved in cross-linking according to alternative A, were found to obey this criterion, thus supporting the proposed model. The distance between K201^ERK2^ and K400^PTP-SL^ was found to be 35.5 Å as opposed to the 12.5 Å distance between K201^ERK2^ and K392^PTP-SL^ thus suggesting that K201^ERK2^ is cross-linked to K392^PTP-SL^ and not to K400^PTP-SL^. Thus, alternative D with K201^ERK2^ linked to K392^PTP-SL^ was found as the most probable scheme of cross-linked residues and distance constraints obeying this network were further used in model building.

**Table 1:** Potential assignments for peptides obtained by tryptic digestion of cross-linked complex between PTP-SL and ERK2.

Observed mass [M+H]^+^	Predicted mass [M+H]^+^	Residue assignment		Sequence assignment	
		PTP-SL	ERK-2	PTP-SL	ERK-2
2221.75	2221.48	*301–312*	*-*	*EIDIPRHGTK^310^NR(2)*	
		*388–392*		*EK^389^NEK(1)*	
	2221.48	*153–157*	*-*	*QDK^155^EK(1)*	-
		*301–312*		*EIDIPRHGTK^310^NR(2)*	
2573.88	2573.97	153–157	147–162	QDK ^155^EK(1)	DLK ^149^PSNLLLNTTCDLK
	2573.97	388–392	147–162	EK ^389^NEK(1)	DLK ^149^PSNLLLNTTCDLK
3155.87	3155.61	*301–312*	-	*EIDIPRHGTK^310^NR(2)*	-
		*390–401*		*NEK^392^CVLYWoxPEK^400^R(2)*	
	3155.71	390–401	193–205	NEK ^392^CVLYWPEK ^400^R(2)	APEIMLNSK ^201^GYTK(1)
3617.27	3617.15	*153–168*	*-*	*QDK^155^EKNQEIHLSPIAR(2)*	-
		*311–323*		*NRYK^314^TILPNPLSR(2)*	
	3617.31	313–329	329–340	YK ^314^TILPNPLSRVCLRPK(2)	FDMELDDLPK ^338^EK(1)

Sequence data corresponding to peptides formed by intramolecular cross-linking of PTP-SL are written in italics. Lysine residues potentially involved in cross-linking through BS^3^ are underlined (numbering is mentioned as superscript). “Wox ”denotes the oxidized form of tryptophan. “1” and “2” between parantheses refer to the number of predicted missed cleavage sites.

### Gel filtration experiments to evaluate the possibility of homomolecular interactions in PTP-SL

According to the potential assignments given in Table 1, homomolecular interactions may take place between PTP-SL molecules. This hypothesis deserves a detailed analysis, since our previous paper reported that the catalytic domain of PTP-SL is a monomer, both in solution and in the crystal structure [14]. In addition, the MW of 120–160, estimated in SDS-PAGE for the complex between ERK2 and PTP-SL, raised the question whether one of the partners within the complex is a dimer. To address this question, we performed gel filtration experiments to determine the MWs of the full cytoplasmic region of PTP-SL (used within the cross-linking experiments), the N-terminally truncated form containing the region 212–549, as well as the catalytically inactive C480S mutant of the full cytoplasmic PTP-SL. Notably, besides the catalytic domain the 212–549 truncated form still contains, the KIM region which is essential for the interaction of PTP-SL with ERK2. Interestingly, while this truncated form displayed an apparent MW practically identical to the theoretical one, the full cytoplasmic PTP-SL eluted with an apparent MW which is 1.74 times higher than the theoretical value (Table 2). A similar MW is displayed by the C480S mutant of this protein. These findings suggest that full cytoplasmic PTP-SL may be a dimer in solution and that dimerization does not involve the active site cysteine C480. The presence of a specific signal at the *m/z* value of 91634.6 in the mass spectrum of the cross-linked complex (FIG. 3A) supports the idea that PTP-SL is a dimer which co-eluted with the cross-linked complex. Full cytoplasmic PTP-SL (both the wild type and the C480S mutant) forms complexes with ERK2 which also display higher MWs than similar complexes based on the truncated PTP-SL 212–549 construct. Considering that the complex of non-truncated PTP-SL contains one ERK2 molecule and two PTP-SL molecules, the experimentally determined MW represents 84% of the theoretical value of this heterotrimer. Thus, gel filtration experiments suggest that PTP-SL containing the full cytoplasmic region, as such and in complex with ERK2, may be a mixture of monomers and dimers.

**Table 2:** Experimental and theoretical MWs of various PTP-SL constructs and of their complexes with ERK2.

Protein	Predicted MW	Observed MW	Observed MW/Predicted MW
PTP-SL 212–549	38207.85	38259.1	1.00
PTP-SL 147–549	45623.15	79394.2	1.74
PTP-SL 147–549 (C480S)	45607.09	80722.5	1.77
Complex of PTP-SL 212–549 with ERK2	80480.45	60883.0	0.76
Complex of PTP-SL 147–549 with ERK2	87895.75	100154.2	1.14
Complex of PTP-SL 147–549 (C480S) with ERK2	87879.69	101829.9	1.16

Experimental MWs were calculated based on gel filtration experiments performed on a Superdex 200 HR analytical column run with mentioned protein preparations under similar experimental conditions (see Materials and Methods).

### Calorimetric study of the ERK2/PTP-SL interaction

**Figure 5:**
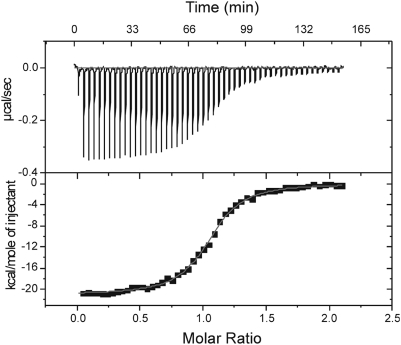
Isothermal Titration Calorimetry. The thermogram (upper panel) and the binding isotherm of the titration of PTP-SLm by ERK2 at 30°C. The thermogram was best fitted to a single site interaction model with a stoichiometry of 1∶1.

In order to get additional elements concerning the stoichiometry of the complex, isothermal titration calorimetric (ITC) measurements were also performed. Figure 5 shows the thermogram and the binding isotherm corresponding to the titration of the inactive mutant (containing active site Cys mutated to Ser) PTP-SL^m^ (9 µM) by ERK2 (75 µM). The isotherm could be best fitted to a single-site binding model, resulting in a stoichiometry of 1∶1 (n = 1.06±0.005), an affinity of K_a_ = 6.6±0.5×10^6^ M^−1^ (K_d_ = 0.15 µM) and a reaction enthalpy ΔH = −21.0±0.1 kcal/mol. From the experimentally determined changes in enthalpy and free energy upon the protein/protein interaction, one can calculate the entropy contribution, TΔS = −11.5 kcal/mol. This negative value means that the entropy term is unfavourable to the interaction, likely due to a decreased conformational freedom of the two proteins upon binding. The intermolecular interaction is therefore driven by the enthalpic contribution. Similar results were obtained when the wild type PTP-SL was used in the ITC experiments.

### Model building with experimental constraints

**Figure 6:**
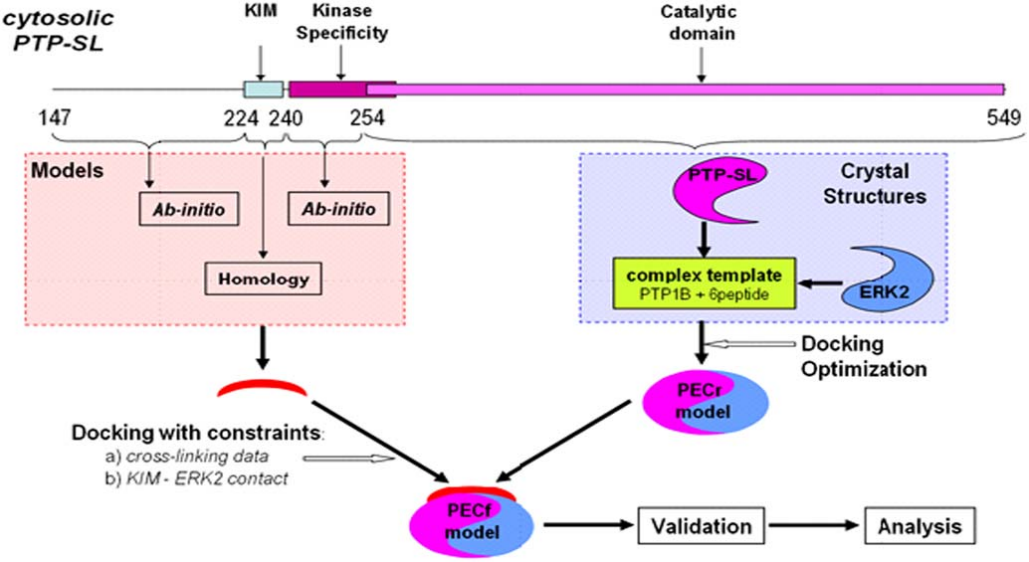
The flowchart of ERK2−PTP-SL complex model building.

The overall model of PTP-SL - ERK2 complex was generated in two steps. First, a model restricted to the regions (aa 254–549) of PTP-SL and (aa 6–358) of ERK2 was generated, starting from the existing crystal structures (PDB codes 1JLN and 2ERK, respectively). This was named PECr. In the second step the PECr model was used to build the rest of the PTP-SL cytoplasmic domain (aa147–253). This was performed by *ab-initio* modeling subjected to all the existing experimental constraints known to date, including those derived from cross-linking experiments (FIG. 6).

**Figure 7:**
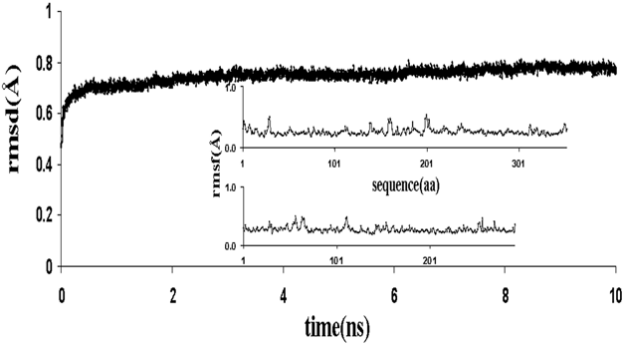
MD simulation RMSD and RMSF profiles. Over a 10 ns span the RMSD remains very low, below 0.75 Å for trace alpha carbons of the PECr complex. The RMSF profile over the sequence, shown in the inset, indicates that the RMSD is mainly due to the loop flexibility.

For building the PECr model, we proceeded as follows. The PTP-1B complexed with a hexapeptide substrate containing phosphotyrosine (PDB code 1PTU), was used as template. On this template, both PTP-SL (aa 254–549) and the ‘new’ substrate, the phosphorylated ERK2, were superimposed, in order to orient the ERK2 in the phosphorylation region similarly to the hexapeptide substrate in the template. This gives the triangulation of ERK2 with respect to PTP-SL. In order to find the best surface of complementarity of the two protein surfaces and to eliminate all steric conflicts, this structure was used as the starting point for docking optimization using Rosetta Dock with constraints imposed on the distances between the WPD loop of PTP-SL and phosphotyrosine on the lip of ERK2 (residues W444^PTP-SL^-pY185^ERK2^, P445^PTP-SL^ -pY185^ERK2^ and D446^PTP-SL^ -pY185^ERK-2^). Rosetta Dock has the advantage of using explicit flexibility of the side chains and was shown to be one of the best ranked protein-protein docking methods, according to the CAPRI evaluation [20]. The best docked structure was further refined by simulated annealing, as described under “Experimental Procedures”. This produced a substantial stabilization of the PEC model reflected in an additional >30% energy loss. The high stability of refined PEC is also confirmed by MD simulation, which shows that, for an overall 10 ns test run the rms deviation remains almost flat, at very low levels of <0.75 Å for trace alpha carbons and <1.0 Å for all PEC atoms (FIG. 7). These values are at the lower end of those usually observed in MD runs at 300 K started directly from crystal structures [21]. This favorable result may be due to several factors such as: the high content of secondary structures; the good complementarity of the partners and the significant decrease of exposed aminoacids in the complex state; and/or to the MD-SA step used to refine PECr which brings the system into a deep local minimum of the energy function. In addition, the overall rms is entirely due to loop flexibility, with no long stretch or domains showing any tendency to significantly deviate from the initial structure in the two proteins (insets in FIG. 7).

It is worth noting here that the PTP-1B-hexapeptide complex (1PTU) was a better template for PECr model than the CDK2-KAP complex [22] for two main reasons. In the first place PTP-SL is 67 aa larger in the N-terminal region than KAP, wich misses helices α0, α1 and α1′ from PTP-SL - and this introduces severe clashes by superposition. The second reason is that ERK2 differs signifficantly from CDK2, both functionally and structurally, in their active site regions. For example, while the rms upstream and downstream the active site between the two phosphatases is less than 1 Å for stretches of over 50aa, for only a 15aa around the active site the rms is over 4 Å, with respect to both the phosphorylated and dephosphorylated forms of ERK2.

**Figure 8:**
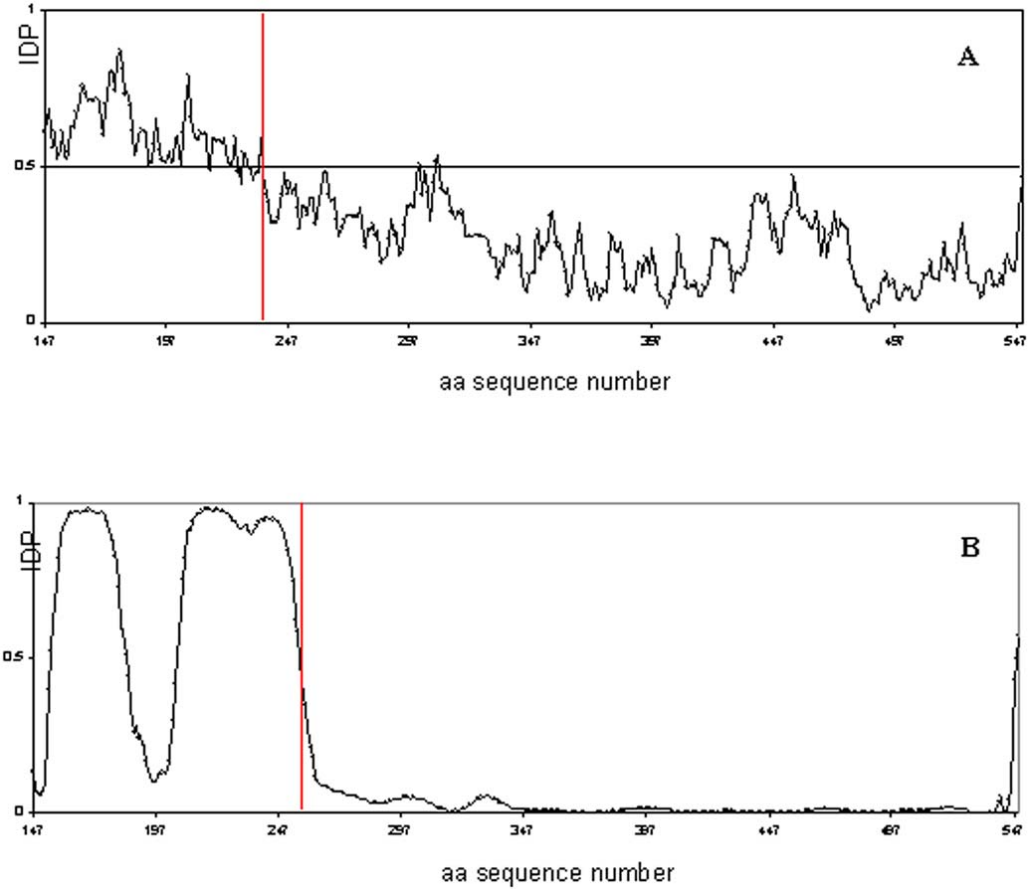
Intrinsic disorder propensity (IDP) of the PTP-SL sequence. IDP was calculated using DISOPRED (A) and IUPRED (B) methods.

**Figure 9:**
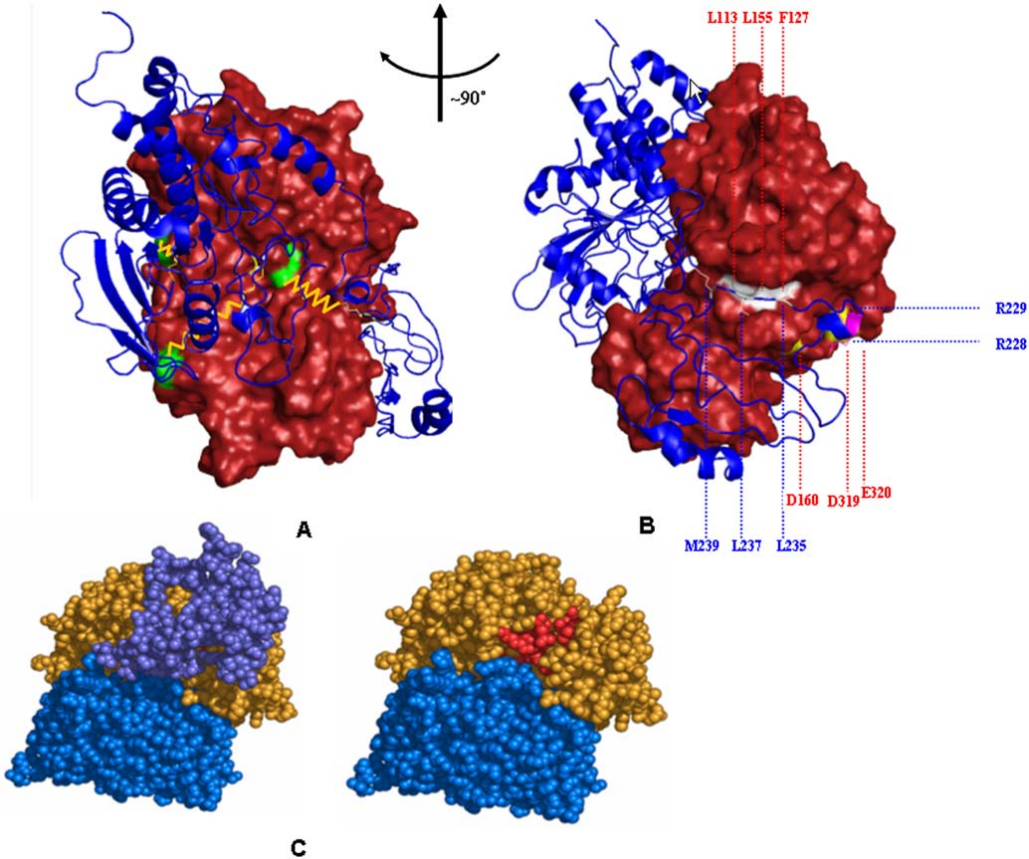
The PECf Model of the overall structure of ERK2−PTP-SL complex in a conformation consistent with phosphatase activity. (A) Network of crosslinks (FIG. 4) within the complex of ERK2 (orange) with PTP-SL (blue). The Lys^PTP-SL^(yellow)-Lys^ERK2^(green) pairs are connected by orange links; (B) Representation of multiple interactions between the KIM containing segment from PTP-SL and ERK2. Cartoon representation: PTP-SL main chain; surface representation: ERK2. The hydrophobic cluster is represented in white; the salt bridges are represented in magenta-yellow pairs. The blue labels designate PTP-SL residues; the red labels designate ERK2 residues. (C) Spacefilling representation of the model of ERK2−PTP-SL complex with and without the segment 147–253^PTP-SL^. Color coding: residues 254–549^PTP-SL^ in blue; residues 147–253^PTP-SL^ in dark magenta; residues of ERK2 involved in ATP binding are colored in red whereas all other residues of the ERK2 chain are in orange.

The PECr model was then used as a starting point to generate the missing region of the cytosolic part of PTP-SL (aa147–253), by combined docking, ab-initio and homology modeling. At present, there are no structural data on this region, as crystallization attempts of the complex have failed so far. Interestingly, this is consistent with predictions resulting from profiling the intrinsic disorder propensity of PTP-SL by two distinct methods (FIG. 8A, B), based either on scores derived from missing coordinates in high resolution protein crystals [23], or pairwise energy estimates from the amino acid composition [24]. Both methods indicate that the PTP-SL region between aa147–250 has a clear tendency for intrinsic disorder. This region might be therefore flexible and consistent with multiple polypeptide paths in various states of the protein. However, given that the interacting KIM motif is located here it is reasonable to presume that the region might become structured in the presence of ERK2. Modeling it in this state by *ab-initio* techniques will yield only a low-resolution structure of rms >5 Å as any prediction for proteins lacking homologues [25]. Nevertheless this low local accuracy can be improved by constraints imposed by various experimental data. The first experimental constraint is supplied by the crystal structure determination of a complex between ERK2 and KIM peptide derived from HePTP (PDB code 2GPH [9] ). The high sequence homology between PTP-SL and HePTP, allowed modeling of this stretch (aa223–253) by homology. The 15 amino acid KIM peptide (aa224–239) was built by co-ordinate transfer and side chain reconstruction while the connection to the PTP-SL (aa254–549) region was built by random loop generation with constraints imposed by the ends and the surface of PEC model, followed by extensive simulated annealing. Finally, the rest of 77 amino acids (aa147–223) had to be modeled by *ab-initio* procedures and to be connected to the PTP-SL (aa224–549) model, both under the docking constraints imposed by the PECr model surface and distance constraints derived from cross-linking experiments and polypeptide connection to the rest of the model. As multiple paths are possible, a repertoire of over 100 *ab-initio* structures was generated with Rosetta. These 3D structures were mapping slightly different PTP-SL stretches of 78–90 amino acids comprising the aa147–223 region, in order to maximize the 3D structural diversity of the *ab-initio* models. Structures were clustered according to their rms deviation and representatives of the top 5 largest clusters were used for docking with constraints imposed by the PEC model surface, the connection at the PTP-SL aa223 end, and the results of cross-linking experiments. From these, the best-matching cross-linking constraints and PEC surface complementarity, was used to complete the overall model of PTP-SL (aa147–549). This final version of PTP-SL (147–549)-ERK2 docking model, named PECf was kept for further structure quality assessment, representation and analysis (FIG. 9). From validation with PROCHECK, the main-chain parameters match those of a crystallographic structure of 2.0–2.5 Å resolution, with a standard deviation of omega angles slightly larger than expected, due probably to CHARMm parametrization. Conversely, the side chain parameters are all better than those of a standard 2.0 Å structure. PROCHECK also shows that over 95% of the residues are in favorable regions of the Ramachandran plot, above the ∼90% limit generally accepted as a threshold for ‘good’ crystallographic models. In addition only a very low fraction of the residues (0.8% in PTP-SL and 1.9% in ERK2) have torsion angles in the fringes of ‘disallowed’ regions, indicating that the structure meets the crystallographic standards at the end of all modeling and simulation steps.

Intermolecular interaction energy analysis at the interface between ERK2 and the low resolution model of the disordered (aa147–253) region of PTP-SL, suggests that the top contribution to the desolvation component of free energy is provided by PTP-SL residues: V182, I205, T209, M220, L225, L235, L237 and M239, while the top contribution to the electrostatic component is provided also by PTP-SL: K155, V190, Q198, R228, R229, E246. Particularly the KIM region seems to contribute significantly to ERK2-PTP-SL complexation in this area of interface.

The PECf model shows that the residues L235, L237, M239 in PTP-SL conserved among the KIM containing D-motif docking peptides, are in van der Waals contact with L113, F127 and L155 residues from ERK2 and form a hydrophobic cluster. This cluster is located near the KIM motif salt bridges, thus making the whole region a strong stabilizing patch of the complex (FIG. 9B). We analysed, the degree of conservation of these three residues within the family of KIM-containing PTPs. We found that aminoacids corresponding to L113 contain all bulky residues and 58.3% of them are hydrophobic. As concerning the positions equivalent to F127 and L155, the degree of conservation of the hydrophobic character is 100% and more than 90%, respectively. Thus, it can be considered that the hydrophobic cluster delineated by L113, F127 and L155 is conserved among the KIM-containing PTPs. Remarkably, this patch is also vicinal to the edge of the ERK2 ATP-binding pocket [8] which, in the present model, is completely covered by the N-terminal part of PTP-SL (aa147–253 - Fig. 9). Thus PTP-SL and ATP may compete in binding ERK2. This finding may explain why ATP is able to dissociate the tight PTP-SL-ERK2 complex [5].

According to the model the PKA phosphorylation site S231^PTP-SL^ is situated on the solvent exposed area of PTP-SL within the KIM region. Although it is not oriented to the interface it is possible that S231^PTP-SL^ phosphorylation will re-orient the side-chain during conformational changes, thus impairing PTP-SL interaction with ERK2 [6].

Our results also indicate that the KIS sequence - immediately following the KIM region and contributing together with KIM to differential binding of PTP-SL to ERK1/2 and p38 - is also involved in specific interactions with ERK2. For instance, residues S281^PTP-SL^ and S282^PTP-SL^ situated on the loop linking α1′ and α2′ helices form hydrogen bonds with S264^ERK2^ situated on the loop α2L14, apparently involved in substrate recognition [8]. Notably, according to this model, the N-terminal half of the KIS region coincides in fact with the flexible linker connecting the KIM region and the catalytic domain of PTP-SL. While S264^ERK2^ is relatively conserved among those MAP kinases where the similar loop α2L14 is present, the tandem serine residues S281–S282^PTP-SL^ are specific for PTP-SL but not for other KIM-containing PTPases. Consequently, this interaction seems to contribute to the mentioned specificity of PTP-SL interaction with ERK2.

Helix α2′ of PTP-SL also plays an important role in interacting ERK2. Thus, F289^PTP-SL^ - part of α2′ – interacts with H230^ERK2^ from L13 through stacking forces and hydrogen bond involving peptide bond of F289^PTP-SL^. The significance of H230^ERK2^ located on L13 resides in its “strategic” position, right before helix G which defines together with helix D, the substrate binding groove of ERK2 [2]. Other residues on loop L13, like P224^ERK2^, P227^ERK2^, K229 ^ERK2^ and Y231 ^ERK2^ also take part in binding PTP-SL at the interface with ERK2. Except for the conserved amino acid P224, the other residues which belong to this loop seem to be rather specific for ERK2 but not for other MAP kinases. On the other hand, the interacting partner residue F289^PTP-SL^ is highly conserved. Experimental findings support the fact that H230^ERK2^ plays a role in substrate binding by ERK2. Thus, H230N mutation, suggested to be a gain-of-function mutation for ERK2 [26], was proved to produce partial resistance to dephosphorylation by phosphatases from HEK293 cells [27].

The model shows that other structural regions of ERK2 responsible for substrate binding, are involved in interface interactions as well. Residues of the loop L12 of ERK2, encompassed between Y185^ERK2^ and W190^ERK2^ and termed “P+1” specificity region, represent such an example. One of the most important of these residues is phosphotyrosine pY185^ERK2^, located on the phosphorylation lip of ERK2. According to our model, its binding to the active site of PTP-SL is assisted by hydrophobic (stacking) interactions with the highly conserved residue Y313^PTP-SL^ which in turn is stabilized through hydrophobic forces between its aromatic ring and the aliphatic portion of the side chain of R312^PTP-SL^. This is an interesting finding, because it confirms previous structural results obtained for the complex of PTP-1B with peptide substrates. Thus, Y46 of PTP1B (equivalent to Y313^PTP-SL^) was found as part of the substrate phosphotyrosine recognition loop [28]. The importance of this residue for the catalytic activity of PTPs is also underlined by the fact that restoration of the equivalent of Y46^PTP1B^ and of the general aspartic acid of the catalytic WPD loop in the catalytically inactive domain D2, confers robust activity in several PTPs [29]–[31]. On the other hand R45 of PTP1B (equivalent to R312^PTP-SL^) is considered a putative substrate binding site [32]. Both R45^PTP1B^ and Y46^PTP1B^ are conserved residues and belong to the NXXKNRY motif, one of the ten conserved motifs which defines the PTP family [32].

The orientation of a phosphorylated hexapeptide interacting with PTP-SL (1PTU) was used as a key constraint in orienting ERK2 with respect to PTP-SL. Thus, PECf conformation does model the complex during ERK2 dephosphorylation. As phosphorylation and dephosphorylation require different conformations [14] and implicitly different interfaces, we examined whether the presence of unphosphorylated form of ERK2 in the complex might induce changes at the interaction interface. Replacing in PECf the phosphorylated ERK2 with its dephosforylated form, taken from 1ERK, results in significant clashes located in three different areas of the interface. As previously reported [2], the activation loop of ERK2 (aa172–189) is shifted extensively in phosphorylated ERK2 adopting distinct conformation as compared to the unphosphorylated form of ERK2. The major consequence of this shift is that these two conformations of the activation loop fall on opposite sides of the WPD catalytic loop of PTP-SL (aa444–449). Therefore, it would be impossible a direct switch by a dynamic trajectory between the two conformations without a significant structural change of the WPD loop in PTP-SL. By contrast, replacing phosphorylated ERK2 in PECf with its dephosphorylated form taken from 2GPH (a structure in which ERK2 is compexed with a KIM peptide analogue), results in practically no clashes - the rms between the two conformations of the ERK2 activation loop being significantly lower - with both structures falling on the same side of the WPD loop. This may suggest that the ERK2 - PTP-SL contact in the KIM region induces in ERK2 conformational changes similar to those corresponding to the phosphorylation of ERK2 activation loop.

## Discussion

Results of the cross-linking experiments, combined with SDS-PAGE and MALDI-TOF analysis of the cross-linked products, as well as the results of gel-filtration experiments on PTP-SL and its complex with ERK2, support the idea that the complex is a mixture of heterodimer and heterotrimer (composed of two PTP-SL and one ERK-2 molecule). By contrast, according to the isothermal calorimetric titration, the stoichiometry of the complex is one PTP-SL to one ERK2 molecule. A possible explanation of these contradictory results might be that the concentration of the complex used in cross-linking and gel-filtration experiments was much higher due to technical constraints, than that used in the ITC experiment. Thus, it may be assumed that in fact an equilibrium between 1∶1 heterodimer and 2∶1 heterotrimer forms exists and high protein concentration shifts the equilibrium towards the formation of heterotrimeric form.

The sub-micromolar range of the dissociation constant of the heterodimer as obtained by calorimetric measurements (0.15 µM) provides a quantitative support for the previously reported experimental finding that this complex involves a very tight interaction between the two proteins[5]. It cannot be ruled out that, under *in vivo* conditions, the complex is in fact a larger entity. Indeed, it has been shown that a member of the PTPBS family of PTPs (human homologues of PTP-SL) forms, with oxysterol-binding protein (OSBP), serine/threonine phosphatase PP2A and cholesterol, a cca. 440 kDa oligomer which regulates phosphorylation of ERK2 in HeLa cells [33]. Taking into account that different residues of ERK2 are involved in interactions with MEK1/2 kinase, PTP-SL and MKP-3 phosphatases [27] it can be imagined that similarly, ERK2 state of phosphorylation is under the coordinated control of both activating and inactivating effectors in the same complex. Dimer state of PTP-SL as such (which can be partially involved in the formation of the complex with ERK2) suggests that dimerization may be part of a mechanism that regulates its enzymatic activity and/or its interaction with specific effectors. The cytoplasmic phosphatase domains of other PTPs, were proved to form homo- and heterodimers; for instance RPTPα forms homodimers [34] and heterodimers [35] with the similar domains of the catalytically inactive IA-2 and IA-2β PTPs, thus down-regulating its enzymatic activity. According to our gel filtration experiments the presence of the N-terminal sequence between L152-S211 is essential for dimerization of PTP-SL. Notably, this sequence roughly represents the first half of the intrinsically disordered region encompassed between aa147–253. Further mutational experiments are necessary to identify the essential residues within this sequence responsible for PTP-SL dimerization.

An interesting principle which seems to govern the interactions between PTP-SL and ERK2, is that regions responsible for substrate binding in individual proteins are reciprocally interacting within the complex, as illustrated by the PECf model. Thus, the KIM, KIS and NXXKNRY motifs from PTP-SL interact with L16, α2L14 and L12 loops of ERK2, respectively, all involved in binding of specific substrates. As concerning the interaction of the L12 loop from ERK2 with KNRY motif of PTP-SL it confirms a former hypothesis that the ERK2 catalyzed phosphorylation and the PTP-SL catalyzed dephosphorylation require different conformations of the complex [14]. The model presented here reflects the dephosphorylating action of PTP-SL. This shows that the “P+1” specificity region from L12 loop of ERK2 binds to R312^PTP-SL^ and Y313^PTP-SL^ from the KNRY substrate recognition loop of PTP-SL, instead of P254^PTP-SL^. Therefore, the phosphorylating activity of ERK2 can be performed only by a rearrangement of the conformation adopted by the complex, in which T253^PTP-SL^ is directed to the active site of ERK2 and consequently, binding of the neighbor residue P254^PTP-SL^ by the “P+1” specificity region on the L12 loop becomes possible. Given that the presence of the KIM region of PTP-SL is essential for complexing ERK2 [4], probably this rearrangement takes place within the complex, while the KIM region is still bound to the docking groove of ERK2. The intrinsic disorder profiles (FIG. 8) show a high flexibility of the S240-P254 linker between the KIM region and the catalytic domain of PTP-SL, supporting thus this hypothesis. The ordered tertiary structure of the whole N-terminal region of PTP-SL (aa147–253), as illustrated by our model, suggests that this intrinsically disordered region of PTP-SL becomes structured in interaction with ERK2. The calorimetric data are in agreement with this interpretation, as the complex formation involves loss of entropy. In other words, the interaction with ERK2 leads to a decrease in flexibility of the N-terminus of PTP-SL within the complex and implicitly, a decrease of entropy.

An important feature of the complex formation between PTP-SL and ERK2 resides in the fact that PTP-SL blocks ERK2 translocation to the nucleus, due to its KIM-dependent binding of ERK2 [5]. Data presented in this paper may provide a structural explanation to this physiologically significant finding. Thus, it is known that activated forms of ERK2 are dimers and dimerization may promote nuclear localization of ERK2 [2], [36]. It has been also demonstrated that ERK2 dimer formation is mediated by a hydrophobic zipper, located on the C-terminal L16 loop [2]. On the other hand, according to our model, PTP-SL interaction with ERK2 involves the same loop L16. Therefore, it can be assumed that the interaction between the phosphorylated ERK2 dimer and PTP-SL leads to disruption of the ERK2 dimer, due to competition for the same L16 region. Consequently, as soon as the ERK2 dimer is disassembled, its translocation to the nucleus may be blocked and ERK2 is retained in the cytoplasm.

PTPRR is a human homologue of PTP-SL and there are reports indicating its involvement in nerve growth factor signaling, thus representing potential target in the therapy of neurodegenerative processes, like Alzheimer disease [37], [38]. Thus, the down-regulating effect of KIM-containing PTPs on ERK2 and on other MAPK activities may play an important role in this respect. Data presented in this paper, as well as the published structure of PTPRR [39], may contribute to a more in-depth understanding of the mechanism of this important regulatory process. In addition, it should be emphasized that the combination of chemical cross-linking with mass spectrometric measurements can be used for the interface analysis of any other protein complexes where only the structures of the partners are known. The reliability of this technique was also demonstrated for the complex between the signal recognition particle and its receptor where the resulting model was in good agreement with crystal structure of the complex [40].

## Materials and Methods

### Plasmid construction, Mutagenesis, Proteins expression and Purification

The cDNA corresponding to the full cytoplasmic region of PTP-SL (aa147–549) and its abridged form (aa212–549) were inserted into the pGEX-4T1 expression vector (GE Healthcare) and the resulting recombinant plasmids were subsequently used to transform DH5α host cells. cDNA of ERK2 was cloned into the pHAT2 expression vector [41] using as host BL21(DE3) cells for the expression of the corresponding protein. The C480S mutant of PTP-SL was obtained by site-directed mutagenesis using QuickChange kit (Stratagene) according to the manufacturer's protocol. All PTP-SL constructs were purified according to the procedure previously described for the catalytic domain of PTP-SL [14]. ERK2 was expressed in pHAT2 as a fusion protein with an N-terminal 6×His-tag. The fusion protein was initially purified by affinity chromatography on a HiTrap Chelating column (GE Healthcare) and then by ion exchange chromatography on a Mono Q column (GE Healthcare). The complexes between PTP-SL constructs and ERK2 were prepared by mixing the purified PTP-SL construct with a slight molecular excess of purified ERK2 protein, with short incubation on ice and finally separation of the complex from the excess of ERK2 by size exclusion chromatography.

### Cross-linking with BS^3^

BS^3^ was obtained from Pierce (Rockford, USA). The PTP-SL- ERK2 complex, in 100 mM HEPES buffer, pH = 7.5 with 300 mM NaCl, was incubated with freshly prepared BS^3^ solution. This solution contains BS^3^ dissolved in 5 mM citrate buffer, pH = 5. Reaction conditions were varied in the following ranges: 0.1–1 mM cross-linking reagent, 2.2–5.54 µg/µl complex, 30–120 min. reaction time and temperature between 0°C and 25°C. The cross-linking reaction was terminated by the addition of 1/6 volume of 300 mM Tris buffer, pH = 7.5. The cross-linking reactions of PTP-SL and ERK2 with BS^3^ were performed under the same optimal conditions as in the case of the complex.

### Gel electrophoresis

Gel electrophoretic separation of cross-linked products and non-cross-linked molecules was performed using 12% polyacrylamide gels under denaturating conditions [42]. MW markers were obtained from Bio-Rad. The protein bands were visualized with Coomassie Blue reagent [42] and Zn/imidazole reagent [43].

### Size exclusion chromatography

To purify the cross-linking conjugate gel filtration was carried out at room temperature with a Superdex 200 HR column 2.2/58. The column was equilibrated with gel filtration buffer: 10 mM Hepes, 150 mM NaCl, 0.1 mM EDTA, 5 mM DTT, 1% SDS, pH = 7.5. A volume of 200 µl cross-linking mixture was incubated for 5 min with 40 µl gel filtration buffer at room temperature, then boiled for 5 min. The boiled sample was centrifuged at 14,000 rpm, 10 min. 220 µl of mixture was applied on the column. Fractions of 500 µl were collected at a flow rate of 0.4 ml/min. A Superdex 200 HR 10/30 analytical column was used in the size exclusion chromatography experiments, to calculate MWs of the PTP-SL forms: 212–549, 147–549, 147–549(C480S) alone and of the various forms of their complexes with ERK2. The gel filtration buffer was the same as previously mentioned but without SDS. For each run 100 µl sample was applied on the column. The flow rate was established at 0.5 ml/min. Gel Filtration Standard (BioRad) was used for MW calibration.

### ITC measurements

Thermodynamic parameters of molecular interactions between ERK2 and PTP-SL at 30°C were investigated by ITC using a MicroCal MCS instrument (MicroCal Inc., Northampton, MA). A PTP-SL solution (8–10 µM) in the calorimeter cell (1.337 mL) was titrated by ERK2 (75–100 µM) using automatic injections of 8–10 µL. The first injection of 2 µl was ignored in the final data analysis. Integration of the peaks corresponding to each injection and correction for the baseline were done using Origin-based software provided by the manufacturer. Fitting of the data to an interaction model results in the stoichiometry (n), equilibrium binding constant (K_a_) and enthalpy of complex formation (ΔH). The experimental data allow calculation of the free energy change (ΔG) and of the entropy term (TΔS), according to the classical thermodynamic formulae: ΔG = −RT×ln K_a_ and ΔG = ΔH−TΔS. All the experiments were repeated twice, and give similar results. The control experiment, consisting in injecting an ERK2 solution into the buffer, shows that the heat of dilution is negligible. The raw calorimetric data were fitted to a simple model with a single type binding site. Three parameters are thus optimized by minimizing the mean square distance between experimental and calculated points. For the stoichiometry n, the optimized value was n = 1.06 with the standard error 0.005, reflecting a great confidence for the 1∶1 stoichiometry. In fact, for affinity values larger than 10^6^ M^−1^ the isothermal binding curve has a clear sigmoidal shape with the inflection point indicating the stoichiometry value.

### MALDI-TOF MS and analysis of MS data

The cross-linked complex purified by size exclusion chromatography (pooled fractions corresponding to 120–160 kDa) was first precipitated with methanol/chloroform according to a reported procedure [44] in order to remove SDS and to increase the protein concentration. Cross-linked PTP-SL, cross-linked ERK2, non-cross-linked PTP-SL and non-cross-linked ERK2, were processed similarly. The pellets thus obtained were then digested with 0.25 mg/ml bovine β-trypsin (sequencing grade, Roche) during 14 h in a digestion buffer containing 100 mM NH_4_HCO_3_, pH 8.2 with 10 mM CaCl_2_. After centrifugation, the supernatants were directly used for MALDI-TOF analysis. The saturated α-cyano-4-hydroxycinnamic matrix was prepared in acetonitrile/aqueous TFA 0.4% (3∶2), mixed with digested samples and applied to a stainless-steel probe plate. Spectra were acquired with a Voyager DE-PRO workstation, using a delayed extraction, linear mode. Mass accuracies were obtained with external calibration. All reported masses are averaged values. The purified, cross-linked complex was also directly analyzed by MALDI-TOF, using sinapinic acid as matrix. The MALDI-TOF spectral data corresponding to the tryptic peptide mixture were introduced in Search-X-links program [18], [19] and analyzed (mass accuracies of ≤120 ppm).

### Modeling

*Homology modeling* was performed with Insight II software package from Accelrys. The Homology module was used for coordinate transfer and loop generation. Local simulated annealing and energy minimization during modeling steps were performed via the Discover module with cvff force field. - *Instrinsic disorder assignment* was performed with DISOPRED [23] and IUPRED [24]. - *Ab initio modeling* was performed using HMMSTR/Rosetta [25] which combines the Monte Carlo fragment insertion for protein tertiary structure prediction (ROSETTA) with the I-sites library of sequence structure motifs and the HMMSTR model for local secondary and supersecondary structure. - *Docking* was performed with InsightII, RosettaDock [45] and CHARMm [46]. InsightII was used to generate the starting coordinates of the complex. Optimization of the binding geometry was performed with RosettaDock with distance constraints. Structure refinement of the complex was performed using the following molecular dynamics simulated annealing (MD-SA) procedure: the MD-SA was started at 1000 K, after an initial 1 ps stage of fast heating the system was cooled down to 10 K at a constant rate of 5 K/0.3 ps. In order to avoid structure distortion during the high temperature steps absolute positional harmonic restraints with a force constant of 50 kcal/molÅ^2^ were imposed on α-carbons of aminoacids along the polypeptide chain found in definite secondary structure states (H, I, E). Simulated annealing was followed by extensive constraint-free energy minimization. - In both MD-SA steps and model stability tests *MD simulation* was carried with CHARMm using CHARMM27 force field [47]. MD runs were performed in explicit solvent using TIP3P water model, with periodic boundary conditions in a 77.6×93.1×71.4 Å^3^ box, at constant temperature (NVT dynamics) at 300 K and RMSD was measured with VMD. Intermolecular free energy calculations were performed with FastContact [48]. Finally the model was evaluated using PROCHECK V.3.4.4 [49] in order to verify if the quality of structure at the end of the modeling procedures meets the crystallographic standards.
